# RB1 gene inactivation by chromothripsis in human retinoblastoma

**DOI:** 10.18632/oncotarget.1686

**Published:** 2014-01-11

**Authors:** Justina McEvoy, Panduka Nagahawatte, David Finkelstein, Jennifer Richards-Yutz, Marcus Valentine, Jing Ma, Charles Mullighan, Guangchun Song, Xiang Chen, Matthew Wilson, Rachel Brennan, Stanley Pounds, Jared Becksfort, Robert Huether, Charles Lu, Robert S Fulton, Lucinda L Fulton, Xin Hong, David J Dooling, Kerri Ochoa, Elaine R Mardis, Richard K. Wilson, John Easton, Jinghui Zhang, James R. Downing, Arupa Ganguly, Michael A Dyer

**Affiliations:** ^1^ Departments of Developmental Neurobiology, St. Jude Children's Research Hospital, Memphis, TN, USA; ^2^ Computational Biology and Bioinformatics, St. Jude Children's Research Hospital, Memphis, TN, USA; ^3^ Biostatistics, St. Jude Children's Research Hospital, Memphis, TN, USA; ^4^ Department of Ophthalmology, University of Tennessee Health Science Center, Memphis, TN; ^5^ Department of Genetics and the; ^6^ Genetic Diagnostic Laboratory at University of Pennsylvania, School of Medicine, Philadelphia, PA, USA; ^7^ The Genome Institute, Washington University School of Medicine in St Louis, St Louis, Missouri, USA; ^8^ Department of Genetics, Washington University School of Medicine in St Louis, St Louis, Missouri, USA; ^9^ Siteman Cancer Center, Washington University School of Medicine in St Louis, St Louis, Missouri, USA; ^10^ Department of Medicine, Washington University School of Medicine in St Louis, St Louis, Missouri, USA; ^11^ Howard Hughes Medical Institute, Chevy Chase, MD; ^12^ Oncology, St. Jude Children's Research Hospital, Memphis, TN, USA; ^13^ Cytogenetics, St. Jude Children's Research Hospital, Memphis, TN, USA; ^14^ Pathology, St. Jude Children's Research Hospital, Memphis, TN, USA; St. Jude Children's Research Hospital – Washington University Pediatric Cancer Genome Project

**Keywords:** chromothripsis, retinoblastoma, RB1, MYCN

## Abstract

Retinoblastoma is a rare childhood cancer of the developing retina. Most retinoblastomas initiate with biallelic inactivation of the *RB1* gene through diverse mechanisms including point mutations, nucleotide insertions, deletions, loss of heterozygosity and promoter hypermethylation. Recently, a novel mechanism of retinoblastoma initiation was proposed. Gallie and colleagues discovered that a small proportion of retinoblastomas lack *RB1* mutations and had *MYCN* amplification [1]. In this study, we identifed recurrent chromosomal, regional and focal genomic lesions in 94 primary retinoblastomas with their matched normal DNA using SNP 6.0 chips. We also analyzed the *RB1* gene mutations and compared the mechanism of *RB1* inactivation to the recurrent copy number variations in the retinoblastoma genome. In addition to the previously described focal amplification of *MYCN* and deletions in *RB1* and *BCOR*, we also identifed recurrent focal amplification of *OTX2*, a transcription factor required for retinal photoreceptor development. We identifed 10 retinoblastomas in our cohort that lacked *RB1* point mutations or indels. We performed whole genome sequencing on those 10 tumors and their corresponding germline DNA. In one of the tumors, the *RB1* gene was unaltered, the *MYCN* gene was amplified and RB1 protein was expressed in the nuclei of the tumor cells. In addition, several tumors had complex patterns of structural variations and we identified 3 tumors with chromothripsis at the *RB1* locus. This is the first report of chromothripsis as a mechanism for *RB*1 gene inactivation in cancer.

## INTRODUCTION

Most retinoblastomas are believed to initiate with biallelic inactivation of the retinoblastoma susceptibility gene (*RB1*) which is rate limiting for tumorigenesis [2, 3]. Over the past 27 years since the *RB1* gene was cloned, researchers have focused on identifying genetic lesions in retinoblastoma that contribute to tumor progression following *RB1* inactivation [4]. Specifically, cytogenetic and array comparative genome hybridization (aCGH) studies have led to the identification of regions of the genome that are gained or lost in retinoblastomas and may contribute to tumorigenesis [4]. Indeed, candidate oncogenes and tumor suppressor genes have been identified whereby copy number variations (CNVs) correlate with changes in gene expression. For example, the *DEK* gene is within the 0.6 Mb minimal region of chromosome 6p22 that is gained in retinoblastoma and there is a significant increase (~2.5 fold) in gene expression in tumors with 6p22 gain (n=5) compared to those without 6p22 gain (n=2) [5]. In a separate study using 21 primary retinoblastomas, Grasemann et al. identified 3 genes *(NUP153, E2F3* and *TTRAP)* with significantly elevated expression (1.7–2.2 fold increase) in tumors with 6p gains [6].

Another recurrent focal amplification found in ~9% of retinoblastomas is a region of the genome on chromosome 2p spanning the *MYCN* oncogene. *MYCN* has been implicated in metastasis in genetically engineered mouse models of retinoblastoma [7] but it is not known if it contributes to progression or metastases in human retinoblastomas. In addition, a recent study reported that a small subset of human unilateral nonfamilial retinoblastomas (1.5%) with *MYCN* amplification (≥10 copies) lack *RB1* mutations. The authors suggested that in those patients, *MYCN* amplification may be sufficient for retinoblastoma tumorigenesis [1].

While aCGH and cytogenetic studies have contributed to the identification of recurrent chromosomal lesions in human retinoblastoma, higher-resolution platforms (i.e. SNP 6.0 arrays) may help to identify additional candidate oncogenes or tumor suppressor genes that contribute to retinoblastoma progression. However, such analyses could be complicated by chromosome instability because it may be difficult to distinguish driver mutations that contribute to retinoblastoma progression from those regions of the genome that are inherently unstable but do not directly contribute to tumorigenesis. Recent whole genome sequencing of 4 primary retinoblastomas and their matched germline DNA demonstrated that at least some retinoblastomas have relatively stable diploid genomes with few CNVs or somatic nucleotide variations (SNV)[8]. Therefore, genome instability may not be required for retinoblastoma progression and the overall low rate of mutation in retinoblastoma may streamline the identification of additional secondary and tertiary genetic lesions in retinoblastomas that contribute to tumorigenesis.

In this study, we performed SNP 6.0 analysis of 94 human retinoblastomas and their matched normal germline DNA. These data allowed us to more precisely define the boundaries of recurrent chromosomal gains and losses and to identify recurrent focal lesions in individual genes or small groups of genes. *MYCN* was the most commonly amplified gene in 8.5% (8/94) of tumors in our retinoblastoma cohort. We also identified focal amplification in *OTX2* in 3% (3/94) retinoblastoma samples. In addition to recurrent deletions in *RB1*, the most common focal deletions were in *BCOR* in 4% (4/94) of our retinoblastomas. To characterize the relationship between *MYCN* amplification and *RB1* gene inactivation, we analyzed the *RB1* gene status in 46 retinoblastomas with sufficient DNA for custom capture Illumina sequencing. Ten of those tumors had no evidence of *RB1* SNVs or indels in the coding region. We performed whole genome sequencing (WGS), RB1 immunohistochemistry and fluorescence *in situ* hybridization (FISH) using probes spanning the *RB1* locus on all 10 tumors. One of the tumors had a wild type *RB1* gene and expressed nuclear RB1 protein in virtually all the tumor cells. It also had *MYCN* amplification consistent with previously published data showing that 1.5% of retinoblastomas may initiate by this mechanism [1]. Most of the tumors had complex structural variations (SVs) inactivating the *RB1* gene and 3 tumors had focal chromothripsis on chromosome 13 spanning the *RB1* locus. This provides a novel mechanism of retinoblastoma initiation and suggests that molecular assays to detect *RB1* chromothripsis should be included in future analyses of this important tumor suppressor pathway.

## RESULTS

### Recurrent Chromosomal Lesions in Retinoblastoma

**Figure 1:**
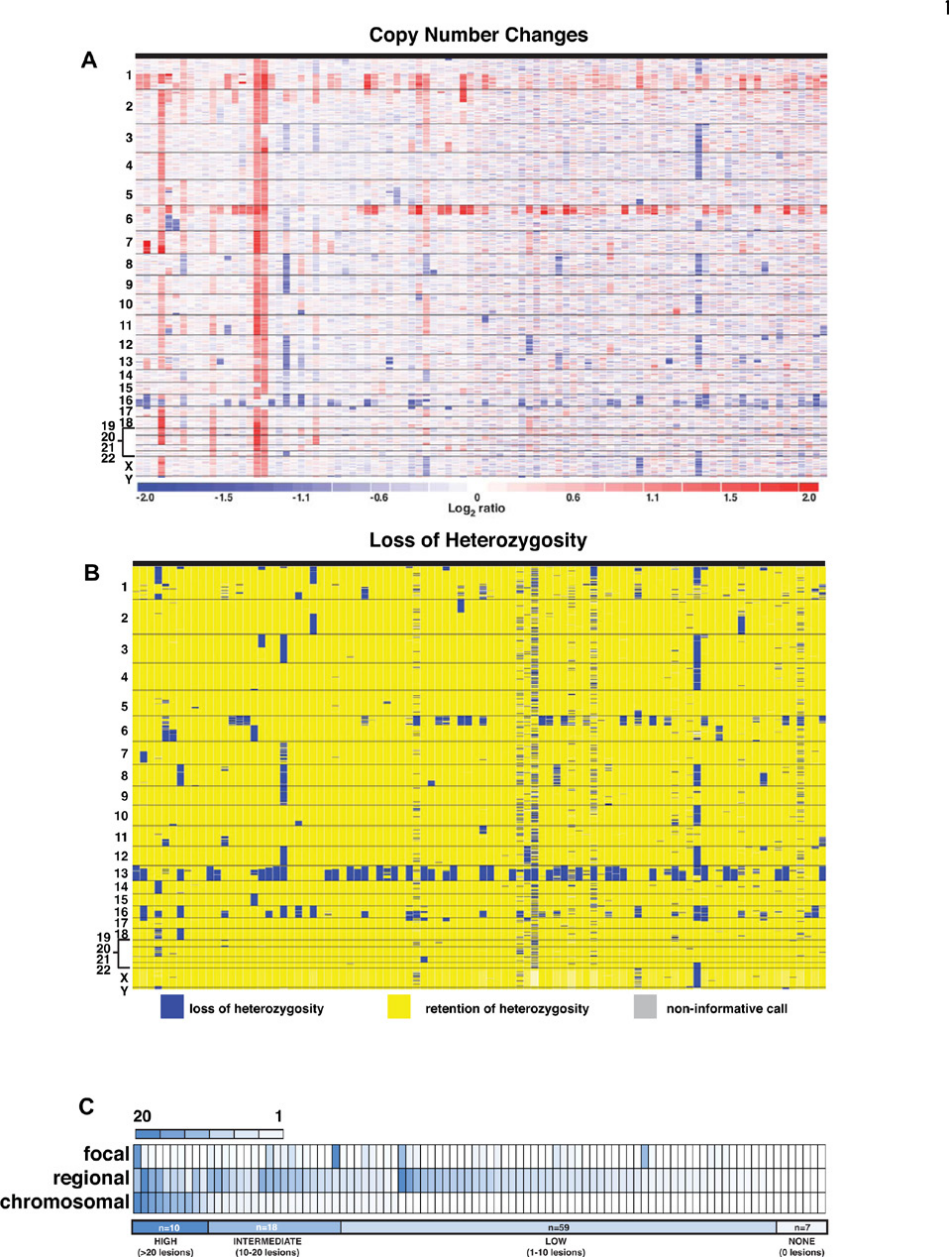
Copy Number Changes and LOH in Human Retinoblastoma Inferred log2 ratio (A) and LOH (B) for DNA isolated from 94 retinoblastomas and matched normal germline tissue. Red is gain and blue is loss in (A). LOH is indicated by blue and yellow indicates no change in genotype in (B). (C) Heatmap of chromosomal, regional and focal lesions for the retinoblastoma cohort. The highest rate of copy number variations (CNVs) was found in 10 tumors (>20 lesions per tumor). An intermediate rate (10–20 lesions per tumor) was found in 18 retinoblastomas and the remaining had low rate of CNV (1–10 lesions per tumor) or none.

Previous aCGH and cytogenetic studies of human retinoblastoma have identified several recurrent whole chromosome gains and losses including chromosome 16 monosomy (18%) [9, 10], gain of chromosome 19 (12–27%) [11] and occasional loss of the X and Y chromosomes[4, 12]. We performed SNP 6.0 analysis of DNA isolated from 94 human retinoblastomas and their matched germline DNA to characterize copy number changes and LOH (Fig. 1 A-C and Table S1). Gains and losses of whole chromosomes were identified in 38.3% (36/94) of retinoblastomas (Tables S2, S3) and 19 of those 36 tumors had a single chromosomal gain or loss (Table S2). In our cohort, the most common whole chromosome losses were chromosomes 16 (12.8%; 12/94), chromosome 8 (6.4%; 6/94) and chromosome X (6.4%; 6/94) (Table 1). The most common whole chromosome gains were chromosome 7 (10.6%; 10/94) and 19 (11.7%; 11/94) (Table 1). The overall average number of gains or losses of whole chromosomes was 1.7 per tumor. In total, 81.9% of retinoblastomas in our cohort had one or fewer whole chromosome gain or loss and only 9.6% had more than 5 whole chromosome gains or losses.

**Table 1:** Frequency of whole chromosome gains and losses in retinoblastoma

Chromosome	% Frequency gain^1^	% Frequency loss^2^	%LOH
1	1.1 (1/94)	0.0	0.0
2	6.4 (6/94)	0.0	0.0
3	3.2 (3/94)	3.2 (3/94)	50.0 (2/4)
4	2.1 (2/94)	2.1 (2/94)	25.0 (1/4)
5	5.3 (5/94)	0.0	0.0
6	6.4 (6/94)	1.1 (1/94)	0.0
7	10.6 (10/94)	0.0	0.0
8	2.1 (2/94)	6.4 (6/94)	30.0 (3/10)
9	4.3 (4/94)	2.1 (2/94)	50.0 (1/2)
10	4.3 (4/94)	1.1 (1/94)	50.0 (1/2)
11	6.4 (6/94)	0.0	0.0
12	3.2 (3/94)	4.3 (4/94)	50.0 (3/6)
13	4.3 (4/94)	4.3 (4/94)	50.0 (2/4)
14	3.2 (3/94)	3.2 (3/94)	0.0
15	2.1 (2/94)	1.1 (1/94)	0.0
16	0.0	12.8 (12/94)	60.0 (9/15)
17	3.2 (3/94)	0.0	0.0
18	6.4 (6/94)	1.1 (1/94)	100.0 (1/1)
19	11.7(11/94)	0.0	0.0
20	8.5 (8/94)	0.0	0.0
21	7.5 (7/94)	0.0	0.0
22	2.1 (2/94)	5.3 (5/94)	20.0 (1/5)
X	4.3 (4/94)	6.4 (6/94)	16.7 (1/6)
Y	4.3 (4/94)	1.1 (1/94)	n/a

1 Whole chromosome gains were defined as 95% of SNP logratios>0 for individual chromosomes.

2 Whole chromosome losses were defined as 95% of SNP <0 for individual chromosomes.

In addition to whole chromosome gains and losses, several whole chromosome arms and/or large chromosomal regions (>3Mb) have been found to be gained or lost in retinoblastoma [4, 12]. In our cohort, we found that 43.6% (41/94) of tumors had chr6p gain, 43.6% (41/94) had a chr1q gain, 12.8% (12/94) had a chr2p gain and 20.2% (19/94) had a chr16q loss (Table S4). We also identified frequent gains and losses of large chromosomal regions (>3Mb) on chromosomes 1, 6, 13 and 16(Table 2). The recurrent gain of *MDM4* in 45% (43/94) on chromosome 1 has been described previously[13–15]. The overall average number of gains or losses of large chromosomal regions (>3Mb), including chromosome arm gains/losses, was 5.2 per tumor (Table S5, S6). In total. 29.8% (28/94) of our retinoblastomas had two or fewer regional chromosomal gains or losses (Table S5, S6).

**Table 2:** Total large regional chromosome lesions (>3Mb)

Chromosome	# of gains	# of losses	Samples^1^	Percent^2^
1	81	16	60	63.8
2	37	2	32	34.0
3	9	4	9	9.6
4	6	3	7	7.4
5	3	9	10	10.6
6	64	5	59	62.8
7	18	1	14	14.9
8	10	7	12	12.8
9	6	3	9	9.6
10	5	5	7	7.4
11	12	6	8	8.5
12	7	4	7	7.4
13	18	26	27	28.7
14	6	4	8	8.5
15	6	0	6	6.4
16	3	31	32	34.0
17	13	14	21	22.3
18	4	3	6	6.4
19	7	4	8	8.5
20	8	1	9	9.6
21	5	0	4	4.3
22	3	3	5	5.3
23	0	1	1	1.1
24	1	2	2	2.1

1 Number of samples with a gain or loss of the indicated chromosome.

2 Percent of samples out of 94 with the indicated chromosome gain or loss.

The number of gains and losses are absolute numbers.

**Figure 2:**
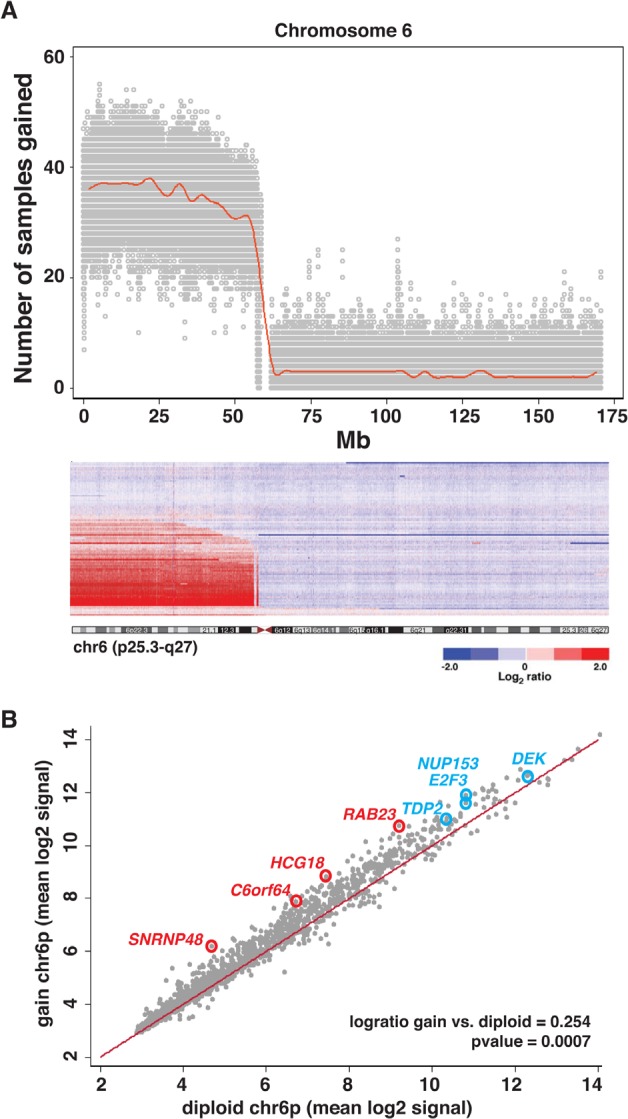
Changes in Gene Expression Associated with 6p Gain (A) Scatterplot of the number of tumors with large regional gains (>3Mb) spanning chromosome 6p. Plot is overlaid with a median spline (red). Below the scatterplot is the corresponding heat map of the log2 ratio of tumor to normal copy number signal across chromosome 6 for all 94 retinoblastomas. (B) Scatterplot of mean gene expression signal for chromosome 6p genes in tumor samples with a gain of chromosome 6p and tumors that are wild type for 6p (diploid). The red line is the unity line where x=y. The genes highlighted with red circles are those that are significantly increased in their expression and have at least a 2-fold upregulation in the tumors with 6p gain. Genes previously identified with increased expression correlated with 6p gain are highlighted with blue circles nuclear ribonucleoprotein (U11/U12). Similar analysis was performed for 1q, 16q, and 2p (Fig. S1). Only 1 gene (*COX4I1*) was differentially expressed in tumors with a 16q loss and no genes were identified on 1q or 2p. The *COX4I1* gene encodes a subunit of cytochrome c oxidase and there is some evidence that this gene is downregulated in skin cancer[20].

To determine if there was any correlation with the most frequent regional chromosomal gains or losses and expression of genes located in those regions, we analyzed gene expression array data from 23 tumors in our cohort that were either diploid or had a gain or loss of the entire arm for chr6p, 1q, 16q, and/or 2p. Overall mean expression level for all genes located on 6p were modestly increased in tumors with a 6p gain (0.254 logratio; p value = .0007). We identified 4 genes on chromosome 6p *(RAB23, HCG18, C6orf64*, and *SNRNP48*) that had statistically significant increase in expression and were more that 2-fold increased in tumors with a 6p gain (p value ≤ 0.05; FDR value ≤ 0.05) (Fig. 2, Table S7). *RAB23* is a downstream effector of Hedgehog signaling in a variety of cancers including those of the bladder, lung and liver[16–19]. *HCG18* is a HLA complex group 18 non-protein coding gene, *C6orf64* encodes an SAYSVFN motif containing protein and *SNRNP48* encodes a small

### Recurrent Focal Lesions in Retinoblastoma

**Figure 3:**
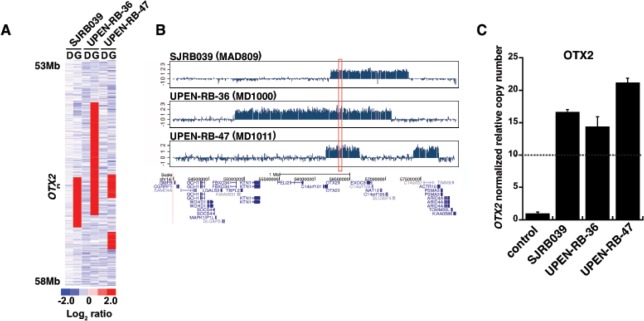
Amplification of OTX2 in Retinoblastomas (A) Heat map and (B) Manhattan plot of inferred log2 ratio of copy number for germline (G) and diagnostic (D) DNA samples of 3 out of 94 samples that carry an amplification in *OTX2* detected by SNP6.0 array analysis. (C) *OTX2* amplification was validated by quantitative real-time PCR. All data was normalized to *CTNNA3* with normal diploid copy number. The dashed line is the cutoff for amplification ≥ 10 copies relative to matched normal DNA for that sample.

To identify recurrent focal lesions in retinoblastoma. we analyzed our SNP 6.0 data for lesions <3 Mb. Among those recurrent lesions that were less than 3 Mb, we defined the minimal region of the individual overlapping lesions, identified recurrent genes in the region and then ranked them by frequency in our retinoblastoma cohort (Tables 3, S8). There were 21 recurrent focal chromosomal lesions (7 gains and 15 losses) in our cohort of 94 tumors (Table 3). Among those, 3 have been reported previously *(RB1* loss, *MYCN* gain/amplification and *BCOR* loss) (Table 3) [3, 8, 21]. The majority (11/16) of recurrent focal chromosomal losses were on chromosome 13 spanning the *RB1* locus (Table 3). The most common focal gain or amplification other than *MYCN* was in a region on chrl4q22.3 spanning *OTX2* (3%;3/94) (Table 3 and Fig. 3). *OTX2* is a homeodomain-containing transcription factor required for retinal photoreceptor development [22, 23]. This focal lesion was validated as an amplification (≥10 copies) by genomic DNA qPCR in each of the 3 samples (Fig. 3C). The most common focal deletion that was not on chromosome 13 spanning the *RB1* locus was in *BCOR* in 4%(4/94) (Table 3). These data are consistent with previous studies showing that *BCOR* is recurrently mutated in retinoblastoma [8]. Taken together, our data suggest that *MYCN* and *OTX2* are the most common focal gains found in retinoblastoma and *RB1*, and *BCOR* are the most common focal losses found in retinoblastoma.

**Table 3:** Recurrent focal gains and losses in retinoblastoma

Gene(s)	Chromosome	Start	End	Change	Frequency
MYCN	2	15858399	16135004	gain	8.5% (8/94)*
OTX2	14	56230461	56634278	gain	3.2% (3/94)*
LOC400794, LRRC52, MGST3	1	163688707	163895545	gain	2.1% (2/94)
DDAH1	1	85752938	85775754	gain	2.1% (2/94)
TRIB2	2	12783000	13014865	gain	2.1% (2/94)
NRG1	8	32390456	32611547	gain	2.1% (2/94)
THSD1, VPS6	13	51876883	51918965	gain	2.1% (2/94)
RB1	13	47797220	47844420	loss	11.7% (11/94)
FNDC3A	13	48414463	48533000	loss	5.3% (5/94)
BCOR	X	39803306	39823169	loss	4.2% (4/94)
CAB39, SETDB2, PHF11, RCBTB1	13	48802186	49077000	loss	3.2% (3/94)
TSC22D1	13	43872987	44057481	loss	3.2% (3/94)
PDCH9	13	66287759	66301124	loss	3.2% (3/94)
RPS6KA1, MTR1976, ARID1A, PIGV, ZDHHC18, GPN2, GPATCH3, NROB2, NUDC,C10RF172, TRNP1, FAM46B, SLC9A1, WDTC1, SYTL1, GPR3, WASF2, FCN3, CD164L2, TMEM222, LOC644961, MAP3K6	1	26690359	27638250	loss	2.1% (2/94)
BTNL9 OR2V2 TRIM7 TRIM41 GNB2L1 TRIM52	5	180375035 180722914 loss	2.1% (2/94)
DIAPH3	13	59564225 59578375 loss	2.1% (2/94)
MIR1369	13	60624885 60691388 loss	2.1% (2/94)
CTAGE11PTBC1D4	13	74100537 74774752 loss	2.1% (2/94)
RNASEH2B-AS1	13	50354743 50367799 loss	2.1% (2/94)
ATP7, ALG11, NEK5, NEK3, UTP14C, MRPS31P5, THSD1, UPS36, CKAP2, TPTE2P3, HNRNPA1L2, SUGT1, LECT1, MIR759, PCDH8, OLFM4, LINC00558	13	51442806	52303952	loss	2.1% (2/94)
RBFOX1	16	6049328	6737903	loss	2.1% (2/94)
CREBBP, ADCY9, SRL	16	3845559	4240638	loss	2.1% (2/94)

* validated by qPCR as amplifications (>10 copies)

### MYCN amplification in Retinoblastoma

**Figure 4:**
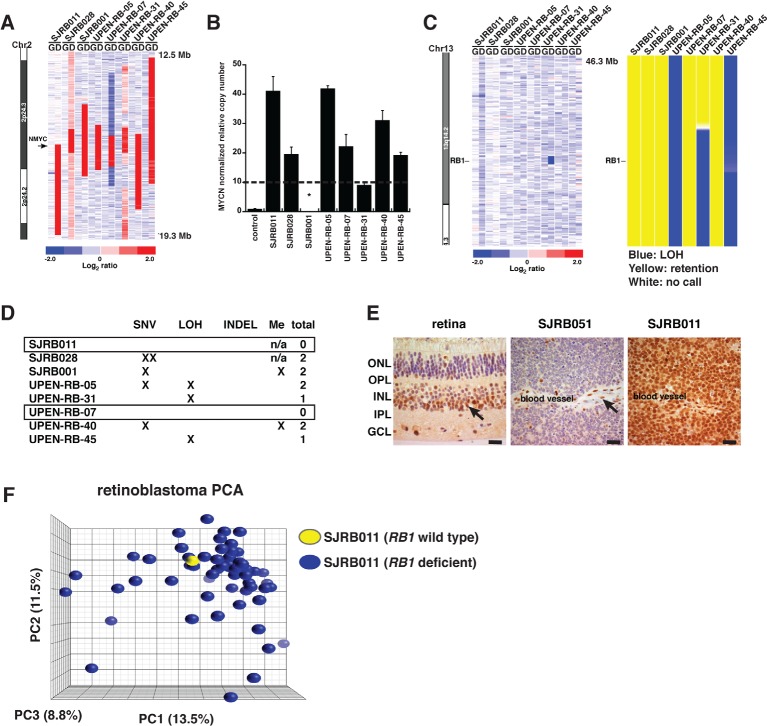
RB1 Gene Analysis in Retinoblastomas with MYCN Amplification (A) Inferred log2 ratio normalized signal for germline and diagnostic DNA samples for samples with *MYCN* gene amplification. (B) One of the samples indicated by an (*) was previously validated to have a *MYCN* amplification (SJRB001). Validation of *MYCN* amplification in the remaining 7 samples using quantitative real-time PCR with a cutoff of 10 copies (dashed line). (C) Inferred log2 ratio for tumor and normal samples and loss of heterozygosity (LOH) for the *RB1* gene as measured by SNP 6.0 analysis. (D) Table summarizing single nucleotide variations (SNV), loss of heterozygosity (LOH), promoter hypermethylation and insertions/deletions (INDEL) at the *RB1* locus. (E) Immunohistochemistry for RB1 in normal retina, SJRB051 with biallelic *RB1* loss and SJRB011 with wild type *RB1*. Arrows indicate RB1 immunopositive cells in the normal retina and vascular endothelial cells. (F) PCA plot of gene expression array analysis for the *RB1* wild type sample (SJRBO11, yellow) and *RB1* deficient retinoblastomas (blue). Scale bars in (E): 10μm.

It has been previously reported that a small proportion (1.5%) of retinoblastomas with *MYCN* amplification express wild type *RB1* [1]. It was proposed that *MYCN* amplification is sufficient to initiate retinoblastoma in those patients [1]. In our cohort, we discovered 8 retinoblastomas with *MYCN* amplification (>10 copies; Fig. 4A,B). Among those 8 tumors, 3 had LOH spanning the *RB1* gene (UPEN-RB-05, UPEN-RB-31 and UPEN-RB-45) and one of them (UPEN-RB-31) had a focal *RB1* deletion (Fig. 4C). Subsequent *RB1* gene sequence analysis and promoter methylation analysis showed that 6 of the 8 tumors with *MYCN* amplification had at least 1 hit in the *RB1* gene (Fig. 4D and Materials and Methods). A tissue block was available for one of the tumors with no apparent *RB1* gene mutations (SJRB011) so we performed RB1 immunohistochemistry on that sample (Fig. 4E). We used a retinoblastoma tumor (SJRB051) with confirmed biallelic *RB1* deletion as a negative control (see below) and normal adjacent retinal tissue as a positive control. The SJRB011 retinoblastoma had abundant nuclear RB1 protein in virtually all the tumor cells (Fig. 4E). To determine if there was any difference in the molecular features of this retinoblastoma with wild type *RB1* and *MYCN* amplification, we performed gene expression array analysis. The SJRB011 tumor was indistinguishable from other retinoblastomas in our cohort by gene expression array analysis (Fig. 4F).

To determine if there were additional tumors in our cohort that had wild type *RB1*, we performed custom capture and Illumina sequence analysis of all 27 *RB1* exons in the 46 tumors with sufficient DNA from patients treated at St. Jude Children's Research Hospital (Table S9). In addition to SJRB011, we identified an additional 9 tumors that had no evidence of SNVs or indels in the *RB1* gene. To characterize the genomic landscape of these 9 tumors in addition to SJRB011 in more detail, we performed whole genome sequencing (WGS) of the tumor and matched normal DNA. Using a paired-end-sequencing approach, we generated 3064 Gb of sequence data for the 10 pairs of samples (germline DNA and tumor DNA); 2793 Gb (91%) were successfully mapped to the reference genome (Table S10). The average genome coverage was 42×, and the average exon coverage was 39×; 99% of SNPs detected across all 20 genomes (10 tumor and 10 germline) showed concordance with their corresponding SNP array genotype calls at the same genomic positions (Table S10).

All somatic alterations including sequence mutations and structural variations were experimentally validated by custom-capture technology and Illumina sequencing. We identified 1201 validated somatic sequence mutations and 306 validated SVs across the 10 tumors (Table 4). These included 25 missense or nonsense gene mutations (tier-1) (Table S11), 122 mutations in regulatory regions or evolutionarily conserved regions of the genome (tier-2), 1,019 mutations in nonrepetitive regions of the genome that are not part of tiers 1 or 2 (tier-3) (Tables 4 and S11). The average number of sequence mutations was 120.1 per case (range, 2–558), with 2.5 mutations per case (range, 0–15) resulting in amino acid changes (Table 4 and Table S11). The average number of validated SVs was 30.6 per case (range, 0–96). The estimated mean background mutation rate was 1.25× 10^−7^ per base (range, 1.34×10^−8^ – 2.28×10^−7^) (Table 4). Consistent with our previous whole genome sequencing results [8], 9 of the tumors had very few SNVs (≤5) in coding regions that resulted in aminoacid changes (average of 2.33 SNVs per tumor). However, one tumor carried 15 SNVs in gene coding regions that resulted in amino-acid changes (SJRB031) (Fig. S2).

**Table 4:** Validated mutations in WGS retinoblastoma data

Sample	tierl^1^	tier2	tier3	Total	Indels	SVs	CNV-AMP	CNV-Mb-AMP	CNV-DEL	CNV-Mb-DEL	Total-CNV-Mb
SJRB011	4(2)	12	72	88	0	24	6	72.52	7	146.24	218.77
SJRB014	0	2	23	25	0	11	3	218.39	6	5.42	223.82
SJRB016	0	0	2	2	0	1	0	0	0	0	0
SJRB020	0	6	45	51	0	3	5	136.31	2	243.93	380.25
SJRB024	3(0)	8	73	84	1	28	38	903.47	0	0	903.47
SJRB031	32(15)	59	467	558	0	74	100	2840.21	13	0.49	2840.70
SJRB032	12(5)	17	171	200	0	17	46	2787.49	0	0	2787.49
SJRB035	0	1	22	23	0	0	0	0	0	0	0
SJRB039	7(2)	12	94	113	0	96	48	227.68	75	117.54	345.23
SJRB051	2(1)	5	50	57	0	52	5	250.60	31	11.19	261.79

1 Tier 1 mutations include all mutations in genes including silent mutations and mutations in the 5′ and 3′ UTRs. The number of nonsynonymous mutations that alter the coding sequence are shown in parentheses.

**Figure 5:**
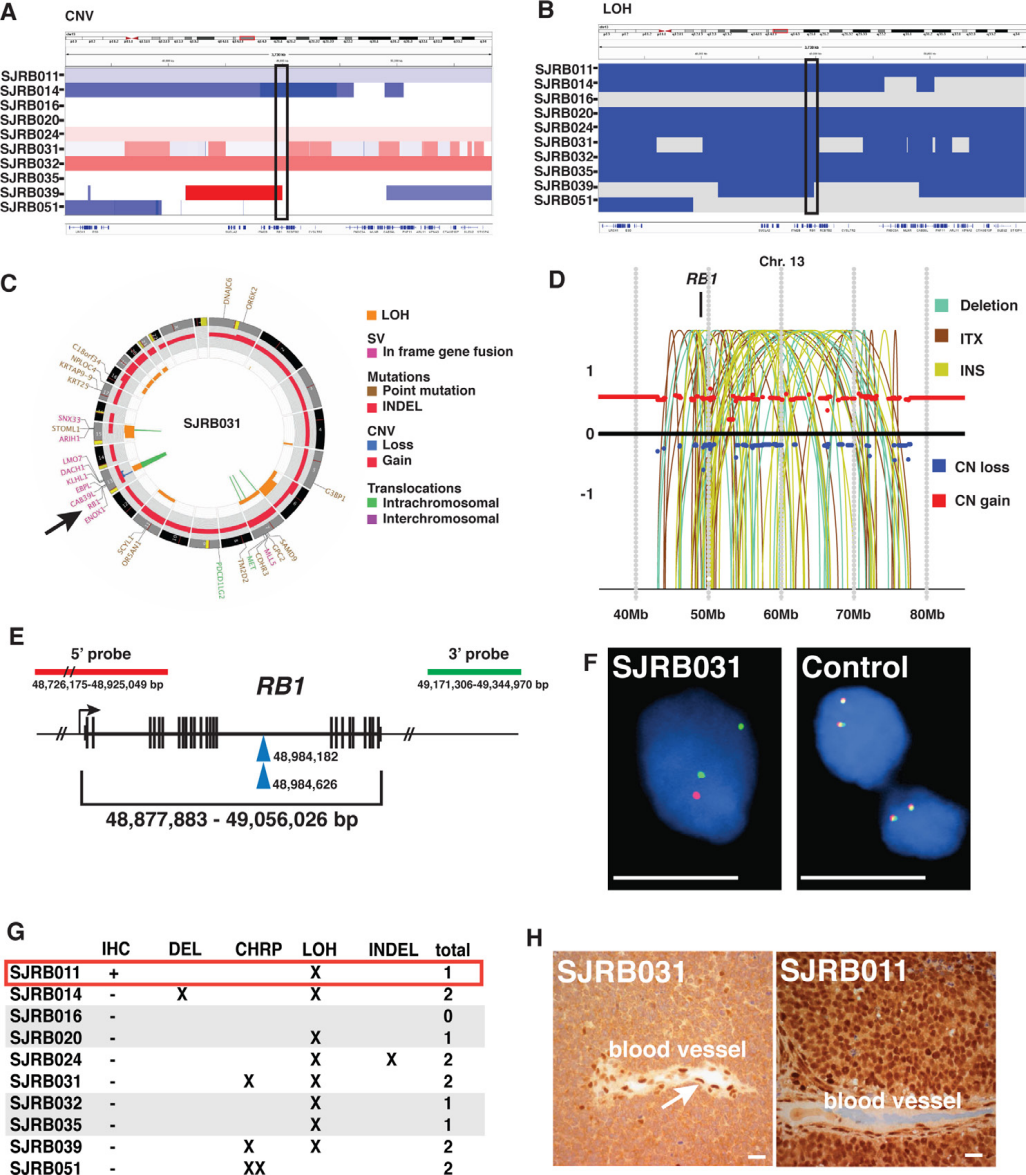
Whole Genome Sequence Analysis of Retinoblastoma (A) Copy number alterations on chromosome 13 spanning the *RB1* gene (box). Blue indicates a loss and red indicates a gain. (B) LOH analysis on chromosome 13 spanning the *RB1* locus (box). Blue indicates LOH and white indicates normal diploid copy number. (C) CIRCOS plot of SJRB031 with chromothripsis at the *RB1* gene (arrow). (D) Half-oval chromothripsis plot of copy number gain (red) and loss (blue) and deletions (gray lines), intrachromosomal translocations (red lines) and insertions (yellow lines). (E) Position of the 5′ and 3′ FISH probes used for 2-color FISH of the *RB1* gene. Blue arrowheads indicate the breakpoints in SJRB031 in the *RB1* gene. (F) FISH for the 5′ (red) and 3′ (green) probes for the *RB1* gene on one sample with chromothripsis (SJRB031) and a normal control (tonsil). (G) Summary of results for immunohistochemistry for RB1 (IHC), deletion analysis (DEL), chromothripsis (CHRP), single nucleotide variation (SNV), loss of heterozygosity (LOH), small insertions/deletions (INDEL) in the *RB1* gene in each of the 10 samples. The red box indicates the one tumor among 94 with wild type *RB1* and expression of RB1 protein. The gray boxes indicate those with one intacti *RB1* gene but no *RB1* protein by IHC. (H) Immunohistochemistry for RB1 in SJRB031. Scale bars in F,H: 10¼m.

Whole genome sequencing confirmed that SJRB011 had a wild type copy of the *RB1* gene even though there was evidence of a reduction in *RB1* gene copy number and LOH spanning the *RB1* locus (Fig. 5A,B). Indeed, 8 of the 10 tumors had LOH spanning the *RB1* gene and 1 of those (SJRB014) had a deletion (Fig. 5A,B). In SJRB024, we identified an indel that led to a frameshift after amino acid 34 of the *RB1* gene (Table S10). This mutation was missed in our *RB1* custom capture and Illumina sequencing analysis due to lack of sequence coverage in this region. Importantly, three tumors (SJRB031, SJRB039, SJRB051) had evidence of chromothripsis on chr13 spanning the *RB1* gene (Fig 5C,D and Fig. S3). The remaining 3 tumors (SJRB016, SJRB020 and SJRB035) did not have any CNVs, indels, deletions, SVs or SNVs in the *RB1* gene from WGS data.

To further validate the chromothripsis and SVs in the *RB1* gene, we developed a two-color FISH assay with separate probes homologous to the 5′ and 3′ regions of the *RB1* locus (Fig. 5E,F). There was agreement between the FISH and WGS data for all 10 samples (Fig. 5E, Figs. S3, S4). For example, in one of the samples with chromothripsis (SJRB031) there was separation of the 5′ and 3′ FISH probes for one allele and the 5′ region of the locus was absent for the other allele (Fig. 5E,F and Fig. S3). To complement the FISH and WGS analysis, we also performed RB1 immunostaining on all 10 samples. Only SJRB011 had nuclear RB1 protein expression (Fig. 4E, 5H and Figs. S3, S4).

## DISCUSSION

In this study, we characterized the chromosomal, regional and focal CNVs and LOH of 94 human retinoblastomas using SNP 6.0 chips. A subset of the tumors (11%) had relatively high rates of chromosomal, regional and focal CNVs with more than 20 lesions per tumor. A larger subset (22%) had an intermediate rate with 10–20 lesions per tumor and the majority of tumors (60%) had few lesions (1–9) or none at all (7%). In addition to the previously reported recurrent focal losses of *RB1* and *BCOR* and amplification of *MYCN*, we also identified a recurrent focal amplification of *OTX2* in 3% of retinoblastomas. We identified 10 tumors in our cohort that lacked *RB1* gene mutations using conventional exon sequencing approaches so we performed whole genome sequence analysis of the 10 tumors and their matched germline tissue. All SNVs, indels and structural variations were validated by custom capture and illumina sequencing. Among those 10 tumors, one had wild type *RB1* with expression of RB1 protein and *MYCN* amplification. One of the tumors had an indel that was not detected in our sequence analysis, one had a deletion and 3 tumors had focal chromothripsis spanning the *RB1* locus and disrupting the *RB1* gene. These data suggest that a regional chromothripsis event may initiate retinoblastoma by inactivating the *RB1* gene.

### The Genomic Landscape of Retinoblastoma

Previously, we performed whole genome sequencing on 4 primary retinoblastomas and their matched germline DNA, as well as an orthotopic xenograft derived from one of the primary tumors that was continuously passaged for 9 months before sequencing [8]. The analysis presented here on 94 tumors validates and extends the previous finding from the whole genome sequencing and earlier aCGH studies. Specifically, the majority (70%) of retinoblastomas have relatively few (≤10 per tumor) chromosomal, regional or focal CNVs. In our cohort, there is no correlation between the rate of CNVs and the heritable or sporadic form of disease nor is there any relationship between the type of *RB1* mutation and the number of lesions. A much larger study will be required to determine if there are more subtle associations between the clinicopathological features of retinoblastoma and the rate of CNVs.

The relatively higher rate of chromosomal, regional and focal lesions in a subset of tumors (30%) does not necessarily indicate that those retinoblastomas have unstable genomes. Chromosome instability is a dynamic process that cannot be accurately measured at a single time point because it involves the acquisition of sequential chromosomal lesions over time. It is important to distinguish between such dynamic processes that reflect the continuous accumulation of genetic lesions from more acute genomic events such as chromosomal “shattering” called chromothripsis [24, 25]. To distinguish the cumulative acquisition of genetic lesions from acute events, it is useful to analyze orthotopic retinoblastoma xenografts over the course of several months in order to more accurately measure the acquisition and selection of mutations.

The overall low rate of CNVs was consistent with the paucity of focal recurrent lesions in genes. As reported previously, we found that inactivation of the *RB1* gene and the *BCOR* gene were the most common deletion events and amplification of the *MYCN* gene was the most common focal recurrent gain[8]. Here we identified a new recurrent focal amplification of *OTX2* in 3% of retinoblastomas. *OTX2* is a homeobox gene that is involved in photoreceptor and retinal pigment epithelium development[23, 26–28]. Retinoblastomas express a variety of rod and cone photoreceptor genes [13, 29] and it will be important to determine if *OTX2* plays a role in modulating the photoreceptor differentiation program in retinoblastoma in future studies.

### Correlations Between CNVs and Gene Expression

For the most common large chromosomal lesions (6p, 1q, 2q, 16q) we integrated our data on copy number changes with the gene expression data from the same tumors. While there was an overall trend of subtle changes in expression for the genes on those altered regions, only 5 genes *(RAB23, HCG18, C6orf64, SNRP48* and *COX4I1)* we found to be altered by more than 2-fold and significantly associated with the copy number alteration. The *RAB23* gene is upregulated in retinoblastomas with 6p gain and there is evidence that *RAB23* may potentiate Hedgehog signaling in cancer [13–16]. Also, the *COX4I1* gene is downregulted in tumors with 16q loss and previous studies have shown that this gene is downregulated in skin cancer [20]. The other genes identified in our analysis have not been previously implicated in cancer.

The genes that have been previously found to show changes in gene expression that correlate with gain of 6p were *DEK, NUP153, E2F3* and *TTRAP*[5, 6]. Those previous studies included fewer samples than the current study and in our larger cohort, those genes did not achieve statistical significance or more than 2-fold changes in expression. We did observe subtle elevation in the expression of *DEK, NUP153, E2F3* and *TTRAP* and additional studies will be required to determine the functional significance of elevated gene expression in human retinoblastoma.

One important caveat of our analysis is the possibility that genes involved in tumor initiation and/or progression may be altered in retinoblastoma irrespective of copy number changes. For example, *MDM4* is found on a region of chromosome 1 that is gained in 45% of retinoblastomas but the gene and protein are increased in virtually all retinoblastomas. Therefore, the lack of a statistically significant association between copy number and gene expression does not necessarily mean that the gene is dispensable for tumorigenesis.

We were not able to perform detailed integrated analysis of gene expression and copy number changes for the less frequent focal lesions such as *MYCN, BCOR*, or *OTX2* because we did not have enough samples with both copy number data and gene expression data. A much larger study will be required to determine if those tumors have distinct gene expression signatures and if there is any association between the focal genetic lesion and the expression of those genes.

### Mechanisms of Retinoblastoma Initiation

One of the goals of our study was to explore the relationship between *MYCN* amplification and *RB1* inactivation to determine if a subset of retinoblastomas can be driven by a single oncogenic lesion *(MYCN* amplification) without *RB1* loss [1]. The possibility of a primary role of *MYCN* in retinoblastoma tumorigenesis was first suggested in 1984 when Lee and colleagues described retinoblastomas with *MYCN* amplifications [21]. However, one of the challenges with identifying retinoblastomas driven exclusively by *MYCN* amplification is excluding all possible mechanisms of *RB1* gene inactivation including SNVs, indels, LOH, deletions, translocations and promoter hypermethylation. In our cohort, we identified 10 retinoblastomas that had no evidence of *RB1* gene inactivation by sequencing the 27 exons of the gene. Whole genome sequencing of those 10 tumors and their matched normal germline DNA showed that at least 5 had mutations. One had an indel that was missed due to poor coverage of that exon in our custom capture sequencing analysis. One had a deletion and LOH that was also missed in our targeted sequence analysis. Remarkably, 3 of the tumors had complex structural variations that occurred focally on chromosome 13 spanning the *RB1* gene locus. These lesions had all the features of chromothripsis and had breakpoints in the *RB1* gene leading to loss of protein expression. This is an important discovery because it is the first report of focal chromothripsis as a mechanism of *RB1* gene inactivation in cancer. Moreover, it suggests that chromothripsis can initiate tumorigenisis by inactivating a tumor suppressor gene. This type of lesion would be missed by conventional *RB1* mutational analysis and this is why it has gone undetected since the *RB1* gene was first cloned in 1986 [30]. Specifically, exon sequencing would return wild type *RB1* sequence because all of the exons are intact in a sample with chromothripsis spanning *RB1*. Copy number and LOH analysis may also show distributions that are difficult to distinguish from the germline reference because both alleles are present and copy number changes are focal and subtle. Our data suggest that a significant proportion of retinoblastomas (3/10) thought to have wild type *RB1* may actually have gene inactivation by chromothripsis. Whole genome sequencing combined with break-apart FISH analysis of the *RB1* locus *and RB1* IHC can be used to identify this unique subset of retinoblastoma tumors.

It is important to emphasize that in our cohort of 10 retinoblastomas that we analyzed by WGS, FISH and IHC there were 5 tumors that had at least 1 intact *RB1* gene (SJRB011, SJRB016, SJRB020, SJRB032, SJRB035). Among those, only SJRB011 had *MYCN* amplification and nuclear expression of RB1 protein. The other 3 had reduced *RB1* protein expression and all 3 lacked *MYCN* amplification. There were no known cancer genes mutated in those 3 tumor samples. It is possible that those tumor samples had biallelic promoter hypermethylation and this caused the reduction in protein expression. Unfortunately, we did not have sufficient DNA to perform methylation analysis. It is also possible that there is a novel oncogenic drive that is altered in those tumors that has not yet been characterized and this in turn leads to downregulation of the RB1 protein. A much larger analysis will be required explore the spectrum of genomic lesions that contribute to retinoblastoma initiation in the absence of *RB1* gene mutation.

## MATERIALS AND METHODS

### Retinoblastoma Tumors Samples

Details for tumors samples acquired at St. Jude Children's Research Hospital have been previously described [13]. In addition, we received DNA for 50 primary tumors and germline samples from the University of Pennsylvania. Tumors and matching blood samples from these patients were identified by clinicians from pediatric oncology clinics within North America and requested the molecular test at the Genetic Diagnostic Laboratory at the University of Pennsylvania. As most of the tested individuals were minors, the respective legal guardians consented. Clinical evaluation of these individuals, and genetic counseling, both before and after the results of genetic testing became available, was provided by the respective referral centers. Genomic DNA was isolated from blood and frozen tumor material using the specific commercial DNA isolation kits following the manufacturer's instructions. Gentra Puregene Blood Kit (3 ml) (Qiagen, P/N 158422) for DNA isolation from blood and DNeasy Blood & Tissue Kit (50) for processing frozen tumor samples (Qiagen, P/N 69504).

### SNP6.0 array assays

Details for the SNP6.0 arrays have been previously described [8]. The SNP6 array data for primary tumors collected at St. Jude Children's Research Hospital were deposited in the dbGaP database (phs000352.vl.pl). Samples were genotyped using Affymetrix SNP 6.0 microarrays according to the manufacturer's instructions. CEL files were generated using GeneChip Command Console Software. SNP calls were generated using Genotyping Console (Affymetrix) and the Birdseed v2 algorithm with default parameters, with at least 50 arrays in each analysis. Array normalization and copy-number inference were performed according to a published workflow[31],[32]. Normalized data were viewed in dChip[33], and regions with abnormal copy number were identified computationally by circular binary segmentation (CBS)[34] and analyzed as described[31],[32]. All calls for gain and loss were manually reviewed as previous described [35] using dChip software [33, 36].

### RB1 mutation analysis

*RB1* mutation analysis was performed as described previously [37]. All 27 exons of *RB1*, including the flanking intronic regions, and the promoter region were amplified by the polymerase chain reaction (PCR) and Sanger sequenced. Large deletions and rearrangements were detected by quantitative-real time PCR assays designed to measure the copy number of each individual exon of *RB1*, including the promoter and 3 ' untranslated regions. Quantitative-real time PCR was performed using a StepOne Plus instrument (Applied Biosystems). Experimental plate setup and analysis was performed in StepOne software v 2.1 (Applied Biosystems). A master mix was prepared for each assay following the manufacturer's instructions. The data was analyzed by the comparative Ct method. The Ct values were then compared with the endogenous control (RNase P) and the reference sample to calculate a ΔΔCt value. Copy number (CN) was then calculated using the formula: CN = 2*2^−^ΔΔCt.

Methylation state of the *RB1* promoter of tumor samples was analyzed using the Methyl-Profiler^™^ DNA Methylation qPCR assays following the manufacturer's instructions, with an assay for RB1 gene (EPHS103757-1A, SABiosciences).

### Gene amplification validation

*MYCN* copy number was detected by a Taqman copy number assay (Hs00824796_cn, Applied Biosystems, Inc.) using quantitative-real time PCR as described above.

*OTX2* copy number was detected by quantitative real-time PCR using Sybr Green (Applied Biosystems 4472942) and analyzed by Eppendorf Mastercycler ep Realplex2 system (Eppendorf, Germany) following manual instruction. DNA was isolated from primary tumor and blood as indicated above. Whole genome amplified DNA was used for samples acquired at St. Jude Children's Research Hospital [8]. Copy number was determined using relative standard curve method. Flanking 5′ and 3′ primers were designed to SNPA-1914401 (rs698015) located in the amplified region of *OTX2* at chr. 14:56339578 and *CTNNA3* designed to SNP_A- 2245588 (rs2105702) at chrl0:67396520 as an internal diploid reference based on SNP6.0 data analysis. Primers were designed using Primer3 [38, 39] for the following:

*OTX2:* forward — AACAGGGCTGGTAAAGAG; reverse - GAGTAGTGCCACTCAGCACA. *CTNNA3:* forward- CAGGTAGGCCAACAAGTCC; reverse — AAGGTACCTGCCATGTGAATA.

### Fluorescence In-Situ Hybridization

The Cancer Center Core Cytogenetic Laboratory received 10 formalin fixed paraffin embedded (FFPE) retinoblastoma tissue specimens in order to determine by FISH if there is a disruption of the *RB1* gene in these tumors. Purified BAC DNA from two *RB1* 3-prime clones (RPH-115122 and RP11-90K7) were labeled with a green-dUTP (AF488, Molecular Probes) by nick translation, and one *RB1* 5-prime clone (RP11-795F23) was labeled with a red-dUTP (AF 594, Molecular Probes). In normal cells that contain normal RB1 gene this probe set produces very tightly linked red and green signals since the probes are separated by only 80kb. This assay was specifically designed to detect any disruption of the *RB1* gene occurring between introns 6 and 16. One hundred interphase nuclei from each tumor were scored for the presence of either normal *RB1* genes (tightly linked red and green signals) or disrupted or deleted RB1 genes (separated red and green signals or deletion of either one). Details for FISH protocol have previously been described [8].

### Immunohistochemistry

Details of IHC protocol have previously been described [13]. IHC was performed on 10 formalin-fixed paraffin-embedded primary tumor samples using 4 μm sections to stain with Retinoblastoma Gene Protein Antibody (clone 13A10- Vector laboratories).

### Whole Genome Sequencing and Exome Capture Validation

Whole genome sequencing and exome capture validation has previously been described [8].

## Supplementary Figures and Tables




