# Supplementary material for: RB1 gene inactivation by chromothripsis in human retinoblastoma
Source: Oncotarget. 2014 Jan 11;5(2):438–50. doi: 10.18632/oncotarget.1686 (PMC3964219; doi:10.18632/oncotarget.1686)
Supplement: Supplementary file 1 [file oncotarget-05-438-s001.pdf]

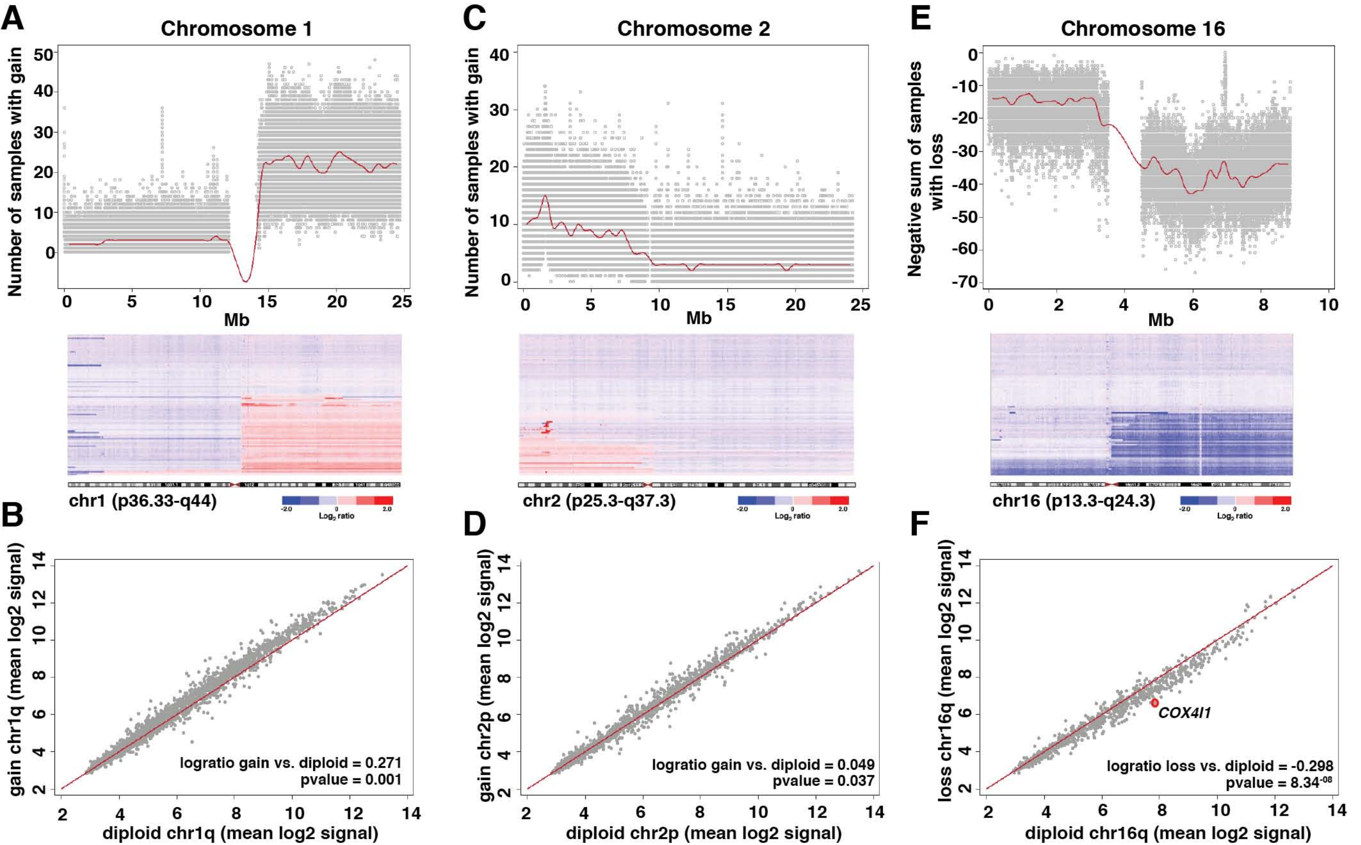
A
Chromosome 1
Number of samples with gain
Mb
chr1 (p36.33-q44)
Log2 ratio
B
gain chr1q (mean log2 signal)
diploid chr1q (mean log2 signal)
logratio gain vs. diploid = 0.271
pvalue = 0.001
C
Chromosome 2
Number of samples with gain
Mb
chr2 (p25.3-q37.3)
Log2 ratio
D
gain chr2p (mean log2 signal)
diploid chr2p (mean log2 signal)
logratio gain vs. diploid = 0.049
pvalue = 0.037
E
Chromosome 16
Negative sum of samples with loss
Mb
chr16 (p13.3-q24.3)
Log2 ratio
F
loss chr16q (mean log2 signal)
diploid chr16q (mean log2 signal)
COX4I1
logratio loss vs. diploid = -0.298
pvalue = 8.34-08

Figure S1. Gene Expression Changes Associated with Gain of Chromosome Arms in Retinoblastoma.

(A,C,E) Scatter plots of the number of tumors with a gain or loss at locations across chromosomes 1, 2, and 16. Plots are overlaid with a median spline (red). Below each is the corresponding heat map of the log2 ratio signal across each chromosome for all 94 retinoblastomas. **(B,D,F)** Scatter plots of mean gene expression signal for genes on each chromosome arm that is entirely gained are lost in retinoblastomas compared to tumors that are diploid. Unity line where x=y (red line). One-sided t-test was used to determine pvalue. One gene is differentially expressed with statistical significance (D) (red circle).

McEvoy_FigS2

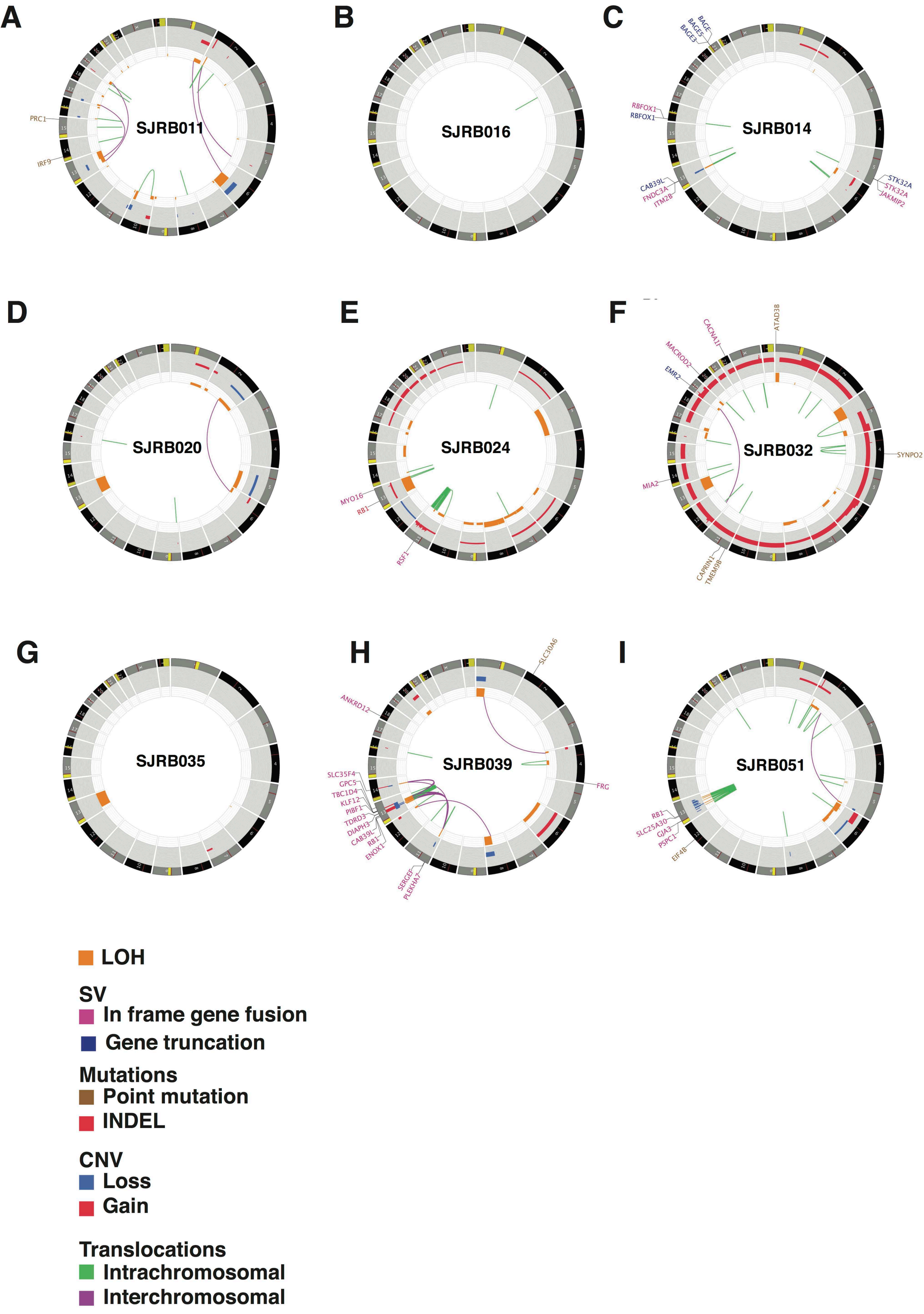

Figure S2. Whole Genome Sequencing of Retinoblastomas.

(A-I) CIRCOS plots of validated mutations and chromosomal lesions in retinoblastomas from our discovery cohort that do not have evidence of SNVs in the *RB1* gene based on custom capture and Illumina sequence. Whole genome sequence analysis identified LOH at the *RB1* locus in all tumors **(A-I),** a single frame shift mutation in the *RB1* gene **(E)**, and structural variations on **(A, C, D, F)** and/or loss of heterozygosity (LOH) (B, E) in the *RB1* gene. LOH (orange), gain (red), and losses (blue) are shown. Intrachromosomal translocations (green lines) and interchromosomal translocations (purple lines) are indicated. Sequence mutations in Refseq genes included silent single nucleotide variants (SNVs, green), nonsense and missense SNVs (brown), splice-site and UTR mutations (dark blue), and insertion/deletion mutations (red).

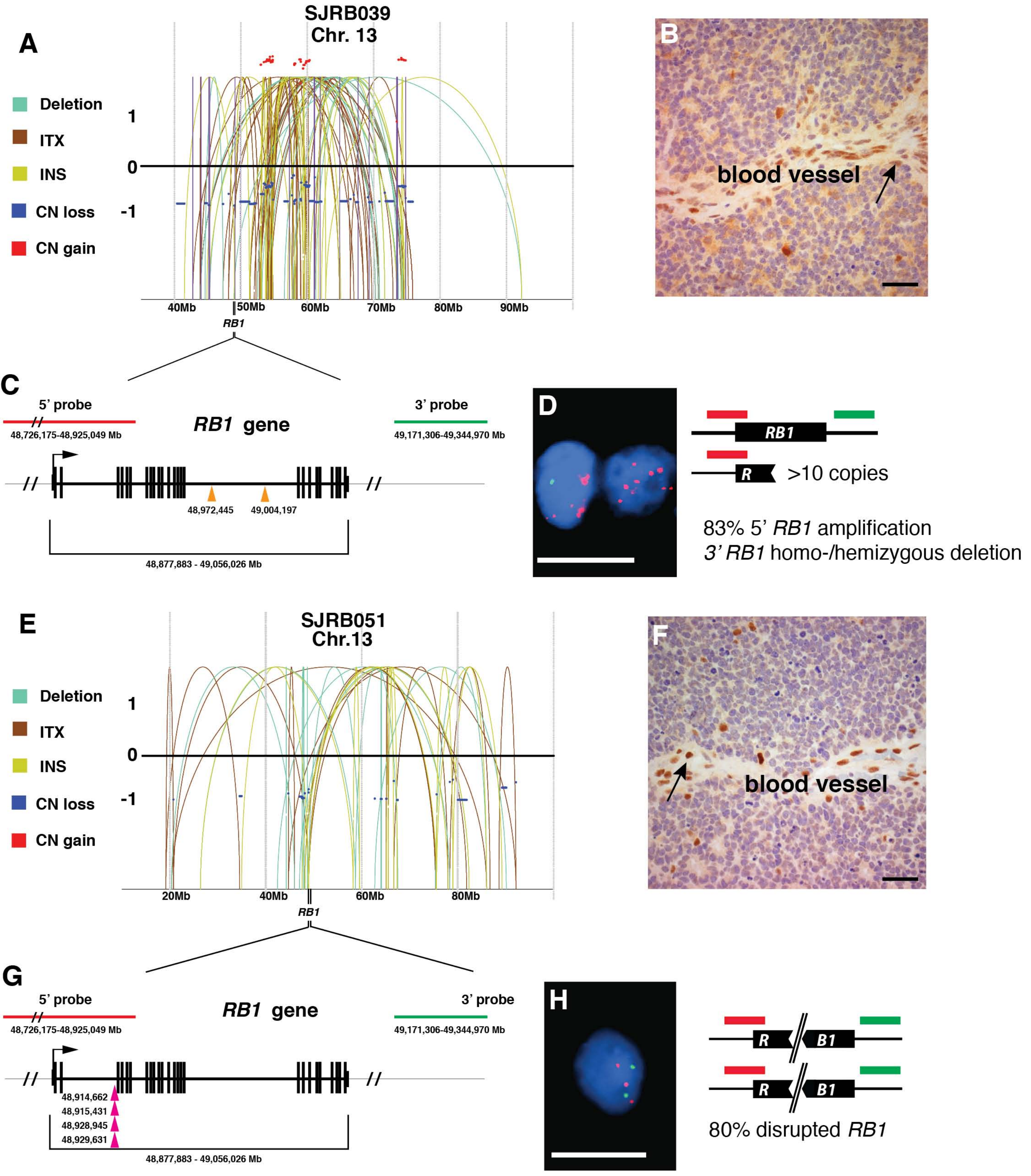
A
SJRB039
Chr. 13
Deletion
ITX
INS
CN loss
CN gain
1
0
-1
40Mb
50Mb
60Mb
70Mb
80Mb
90Mb
RB1
B
blood vessel
C
5' probe
48,726,175-48,925,049 Mb
RB1 gene
3' probe
49,171,306-49,344,970 Mb
48,972,445
49,004,197
48,877,883 - 49,056,026 Mb
D
RB1
R
>10 copies
83% 5' RB1 amplification
3' RB1 homo-/hemizygous deletion
E
SJRB051
Chr.13
Deletion
ITX
INS
CN loss
CN gain
1
0
-1
20Mb
40Mb
60Mb
80Mb
RB1
F
blood vessel
G
5' probe
48,726,175-48,925,049 Mb
RB1 gene
3' probe
49,171,306-49,344,970 Mb
48,914,662
48,915,431
48,928,945
48,929,631
48,877,883 - 49,056,026 Mb
H
R
B1
80% disrupted RB1

Figure S3. Disruption of *RB1* Gene in Retinoblastomas with Chromothripsis

(A) A half oval chromothripsis plot showing interchromosomal translocations, CNV, deletions and insertions on chromosome 13 spanning a ~40Mb region including the *RB1* locus in SJRB039. Copy number gain, loss, or no change is defined along the Y-axis as 1, -1 and 0, respectively. **(B)** Immunohistochemistry for RB1 on SJRB039. The blood vessel with immunopositive vascular endothelial cells is highlighted by the arrow. **(C)** Map of the *RB1* locus and the 5' and 3' FISH probes used to validate the chromothripsis. Validated breakpoints identified in the WGS data are shown as arrowheads in the lower portion of the drawing. **(D)** Representative FISH image for SJRB039 showing separation of the red and green probes corresponding to the 5' and 3' end of the gene, respectively. One hundred interphase nuclei were scored. **(E)** A half oval chromothripsis plot showing interchromosomal translocations, CNV, deletions and insertions on chromosome 13 spanning a ~40Mb region including the *RB1* locus in SJRB051. Copy number gain, loss, or no change is defined along the Y-axis as 1, -1 and 0, respectively. **(F)** Immunohistochemistry for RB1 on SJRB039. The blood vessel with immunopositive vascular endothelial cells is highlighted by the arrow. **(G)** Map of the *RB1* locus and the 5' and 3' FISH probes used to validate the chromothripsis. Validated breakpoints identified in the WGS data are shown as arrowheads in the lower portion of the drawing. **(H)** Representative FISH image for SJRB039 showing separation of the red and green probes corresponding to the 5' and 3' end of the gene, respectively. One hundred interphase nuclei were scored. Scale bar 25μm in B,F and 10μm in D,H.

McEvoy_Fig.S4

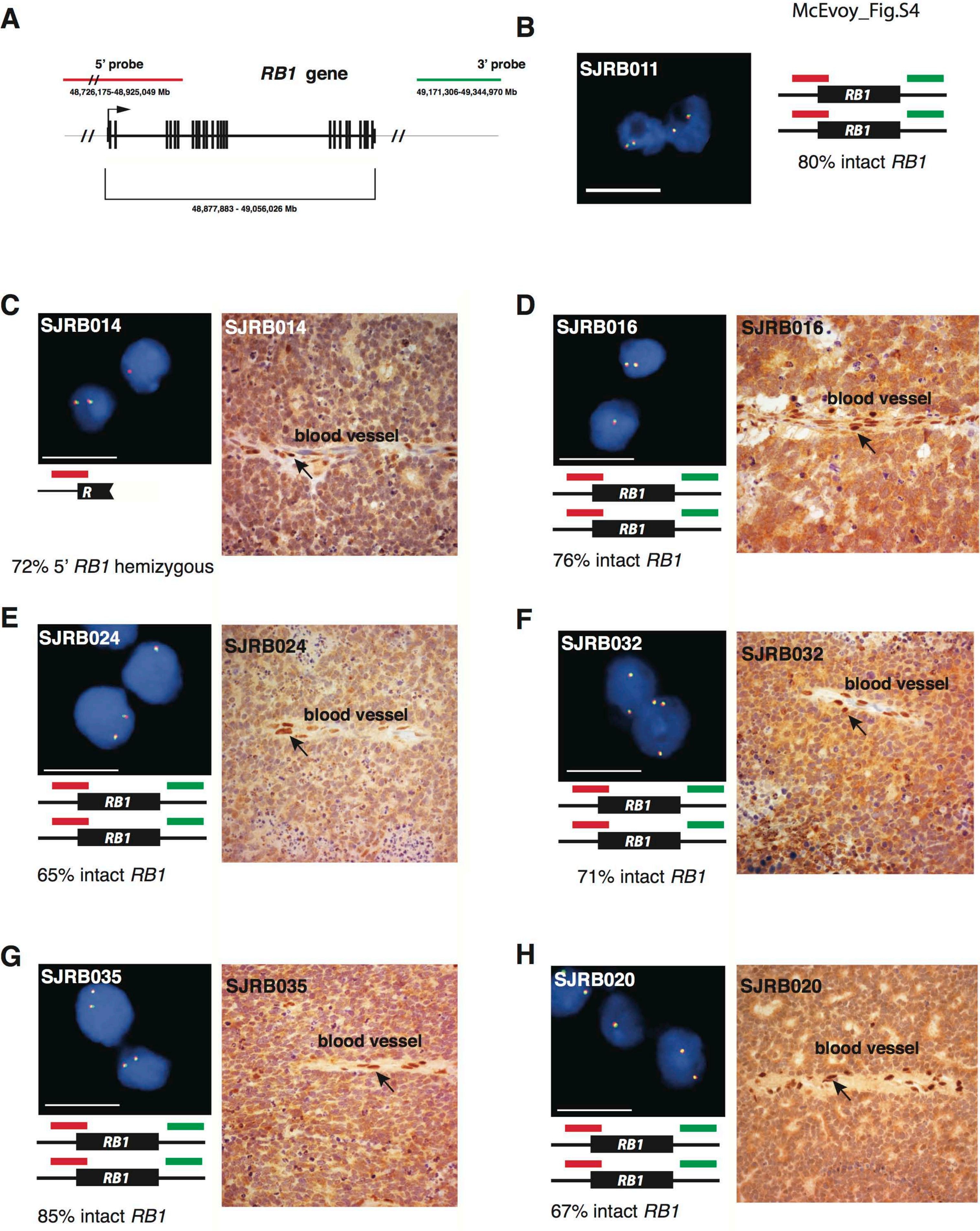

Figure S4. RB1 FISH and IHC. (A) Map of the *RB1* gene and 5' and 3' probes used for 2-color FISH. **(B)** 2-color FISH for SJRHB011 showing co-localization of the 5' and 3' probes that are red and green fluorescence, respectively. A summary drawing of the organization of the *RB1* locus is presented adjacent to the FISH image. **(C-H)** Representative FISH and IHC for each of the samples that were sequenced in this study and were not presented in the main figures or other supplemental figures. A summary drawing of the structure of the *RB1* locus is shown below each FISH image. For the IHC images, the blood vessel is indicated and an RB1 immunopositive cell from the vascular endothelium is highlighted with the arrow. Scale bars: FISH, 10μm IHC, 25μm.
